# Psychological AI: Designing Algorithms Informed by Human Psychology

**DOI:** 10.1177/17456916231180597

**Published:** 2023-07-31

**Authors:** Gerd Gigerenzer

**Affiliations:** Max Planck Institute for Human Development, Berlin, Germany

**Keywords:** heuristics, artificial intelligence, recency, Google Flu Trends, explainable AI

## Abstract

Psychological artificial intelligence (AI) applies insights from psychology to design computer algorithms. Its core domain is decision-making under uncertainty, that is, ill-defined situations that can change in unexpected ways rather than well-defined, stable problems, such as chess and Go. Psychological theories about heuristic processes under uncertainty can provide possible insights. I provide two illustrations. The first shows how recency—the human tendency to rely on the most recent information and ignore base rates—can be built into a simple algorithm that predicts the flu substantially better than did Google Flu Trends’s big-data algorithms. The second uses a result from memory research—the paradoxical effect that making numbers less precise increases recall—in the design of algorithms that predict recidivism. These case studies provide an existence proof that psychological AI can help design efficient and transparent algorithms.

In this article, I address two questions: Is there any use for psychology in the design of artificial-intelligence (AI) algorithms? And if so, what kind of psychological insights are helpful? Like most of AI, the algorithms I am concerned with here attempt to solve the same problems as humans do. The psychological insights I focus on concern what is known about the mind, such as cognition and decision-making, not about the biology and physiology of the brain. Although one might assume that millions of years of evolution of the human mind have generated cognitive processes that would fascinate engineers, the reality shows otherwise.

Looking back in history, it becomes clear that the big successes of AI made little use of psychology. IBM’s Deep Blue, the program that beat Kasparov in 1997, was not based on Herbert Simon’s and colleagues’ earlier psychological research on the thought processes of chess masters (Simon, 1979). When Joe Hoane, one of Deep Blue’s programmers, was asked how much of his work was devoted specifically to AI in emulating human thought, he responded: “It is not an artificial intelligence project in any way. It is a project in—we play chess through sheer speed of calculation and we just shift through the possibilities and we just pick one line” (Krauthammer, 1997). Nevertheless, its evaluation heuristics had been tuned by chess experts—as in the case of chess algorithms, such as Stockfishm but unlike AlphaGo Zero and its descendants, such as Leela Chess Zero, whose eponymous “zero” indicates that no human knowledge has been added. Google Flu Trends (GFT), once hailed as the great success of big-data analytics, analyzed the correlations of some 50 million search terms with indicators of flu and tested 450 million different algorithms to find the best one (Ginsberg et al., 2009). No research on the psychology of prediction was used for building the algorithm. Natural-language models such as GPT-4 estimate billions of parameters from massive amounts of online text, basically counting what passages of words follow other passages to create fluent texts that read as if they derived from a human. Whereas the biology of the brain has inspired aspects of deep artificial-neural networks, insights from the psychology of language, in particular concerning semantics and pragmatics, appear to have had little impact on the creation of these language models. As a final example, deep neural networks have been purported to represent the best models of human vision, yet as Bowers et al. (2022) pointed out, psychological theories—such as Ramachandran’s 1988 work on how shape is inferred from shade by evolved heuristics—have received little consideration in the development of these models. In consequence, despite their success in classifying images and predicting human errors, deep neural networks account for almost no experimental findings in the psychology of vision (Bowers et al., 2022).

A glance at machine-learning textbooks verifies that insights about the human mind are rarely used to inform the design of algorithms. Most textbooks teach nothing but statistical tools (e.g., Hastie et al., 2017); some add a nod to psychology in a page or two that may allude to William James and Frederic Bartlett (Russell & Norvig, 2016). A few delve admirably deep into behaviorist-learning theory (e.g., Sutton & Barto, 2018), but even here, the concern is to point out similarities and differences, not to use psychology explicitly for designing algorithms. Part of the research on the psychology of reasoning has unwittingly supported this neglect by arguing (incorrectly, in my view) that following the rules of logic and Bayesian probability is necessary and sufficient for human rationality. These rules can be executed more precisely and faster by a computer, making psychology appear to be obsolete or even the source of irrationality. Exceptions to this general picture exist. For instance, Ho and Griffiths (2022, Section 3.3) provided examples for how psychological insights could be used to inform the design of algorithms, and Lake et al. (2017) called for including psychological results about intuitive physics and intuitive psychology.

## Origins of Psychological AI

Psychological AI is the use of psychological principles for the design of machine algorithms. As mentioned above, by “psychological principles,” I refer to theories and facts about mental and social processes, such as cognitive heuristics and social organizations, not to the biology and physiology of the brain (which has had its impact). By “design,” I mean the use of these principles in the construction of algorithms, not after-the-fact analysis of similarities. Thus, my focus is not on similarities, such as when von Neumann (1958) considered the possibility of an analogy between the human nervous system and the digital computer, or on insights about brain structures, such as when Lindsay (2021) argued that convolutional neural networks were inspired by the organization of the cells in the visual cortex. Psychological AI also differs from the tools-to-theories heuristic, in which researchers propose familiar statistical tools as theories of mind (Gigerenzer, 1991). Instead, in psychological AI, theories of mind are used to inform the design of algorithmic tools.

Psychological AI is nothing new. In fact, the first machine computers were modeled after a social system. After the French Revolution introduced the decimal system for measuring weight and height, this required calculating new logarithmic and trigonometric tables. Inspired by Adam Smith’s (1776/1937) *The Wealth of Nations*, the engineer Gaspard de Prony did not ask mathematical prodigies to perform the calculations, as was common up to then, but introduced a new three-tiered division of labor. At the top were a few famous mathematicians such as Adrien Legendre, who devised the formulas; in the middle were seven or eight persons trained in analysis; and at the bottom, there were 70 to 80 unskilled workers who simply added and multiplied millions of numbers (Daston, 1994). This human computer calculated 10,000 sine values to the unprecedented precision of 25 decimal places and some 200,000 logarithms to 14 or 15 decimal places. The project, one the world had never seen before, inspired Charles Babbage to replace factory workers with machinery and to build the first mechanical computers. Thus, in the beginning, there was a new social organization of work, and the machine computer was modeled in its image. The very elements of large-scale division of labor, such as subroutines and hierarchical organization, became the essence of digital computers.

When Herbert Simon and Allen Newell tested their first AI program, the logic theorist, they, like Prony, used a computer made of human components, including Simon’s wife, children, and graduate students, who executed the subroutines (Gigerenzer & Goldstein, 1996). Simon’s original program was an early version of psychological AI: to analyze the heuristics that human experts such as chess masters rely on, formalize these, and thereby make computers intelligent.

In what follows, I outline a program of psychological AI, the use of psychological principles to make algorithms smart and transparent. Every program or theory should state its domain and, thus, its limits. I argue that a distinction from decision theory can identify the domain of psychological AI, that is, the types of problems for which the latter is useful or not. Then I discuss the psychological principles that are relevant and provide two illustrations. Finally, I describe the solution that psychological AI offers to the goal of explainable AI.

## The Stable-World Principle

Why can AI easily beat the best human chess players but still struggle with human-created captchas? Why is big data useful for predictions in astronomy but was unable to predict the financial crises of 2008 and Trump’s victory in 2016? One response is to simply wait in anticipation of more computational power and better data that will finally empower algorithms to solve all tasks perfectly, or at least better than humans do. However, computing power is sufficient for only specific kinds of problems.

A more theoretical response can be derived from a distinction in decision theory, that between situations of “risk” and “uncertainty,” which Knight (1921) first identified. A “situation of risk” is a special case of a “small world,” where the set *S* of all future states along with the set *C* of all of their consequences are perfectly known (Savage, 1954/1972). This pair (*S*, *C*) is called a “small world.” A small world with unknown probabilities represents a situation of “ambiguity”; one with known probabilities is a situation of risk. Situations of risk include the stock-in-trade of decision research, such as lotteries and choices between monetary gambles. “Uncertainty,” in contrast, refers to ill-defined situations that are called “large worlds,” where the “state space” (*S*, *C*) is imperfectly known or unknowable because it changes in unexpected ways. Here, no probability distribution (with probabilities that add up to 1) can be meaningfully constructed over states or consequences, not even subjective probabilities. Savage (1954/1972, p. 16) made it clear that the Bayesian theory of expected utility maximization applies only to small worlds and considered it “ridiculous” to apply the theory in situations of uncertainty, be they as mundane as planning a picnic or as intractable as playing chess. Despite Savage’s warning, many psychologists, assuming that Bayesian decision theory defines rationality for all problems, do not distinguish between risk and uncertainty (Volz & Gigerenzer, 2012). The distinction between small and large worlds is related to that between closed-world problems and open-world problems, such as between well-defined and ill-defined games.

When applying the distinction between risk and uncertainty to AI, the “stable-world principle” defines its domain and boundaries (Gigerenzer, 2022, p. 39; see also Katsikopoulos et al., 2020): Complex algorithms work best in well-defined, stable situations in which large amounts of data are available. Human intelligence has evolved to deal with uncertainty independent of whether big or small data are available.

The distinction between risk and uncertainty is often overlooked in psychology. For instance, choice between well-defined gambles, that is, situations of risk, are often referred to as “situations of uncertainty” (e.g., Bourgin et al., 2019). Optimal solutions in situations of risk, however, are not necessarily the best solutions in situation of uncertainty, in which, by definition, the optimal course of action cannot be known (Volz & Gigerenzer, 2012).

That principle makes it clear why AI systems deliver excellent results for some problems but not others. For instance, games such as chess, Go, and Jeopardy! are well-defined, stable situations in which complex algorithms with big data have flourished. In contrast, in the prediction of human behavior, such as recidivism (Dressel & Farid, 2018) and the future of fragile families (Salganik et al., 2020), simple algorithms that rely on a few cues typically perform as well as complex algorithms and with less data. Which particular simple heuristics perform well, in turn, depends on their ecological rationality (see below).

## The Program of Psychological AI

### The domain of psychological AI

The stable-world principle provides a preliminary definition of the domain of psychological AI: Psychological AI uses insights from psychology to design efficient and transparent algorithms for ill-defined and unstable problems.

Unlike in machine learning, the “I” in psychological AI stands for human intelligence. Ill-defined problems and unexpected events characterize situations in which complex algorithms and big data struggle, such as when predicting recidivism, the ideal romantic partner, the futures of families at risk, and human behavior in general (Gigerenzer, 2022). Prediction here means real prediction, that is, forecasting the future under conditions of uncertainty (“out-of-population prediction”), as opposed to “out-of-sample prediction” (which creates a small world by repeatedly sampling from the same population) or mere data fitting (Katsikopoulos et al., 2020). The stable-world principle also provides a hypothesis that psychological AI meets its limits when confronted with well-defined tasks with rules that are stable over time, such as routine industry applications and games such as chess.

Simon’s original program of using psychological principles to make computers smart failed to distinguish between stable and unstable worlds. He and his students studied chess masters at length and asked them to think aloud while playing. In 1958, he predicted that within 10 years, a computer would beat the world’s chess champion (Simon & Newell, 1958). Simon erred not only in terms of the time frame but, more critically, in that the program that beat Kasparov did not win by mimicking human intelligence, as Simon had expected. Here he, like many others, overlooked a fundamental issue, the stable-world principle. Psychological AI revives the original vision of AI promoted by Simon and Newell (1958) but with an important domain qualifier: to deal with ill-defined and unstable problems.

### The tools of psychological AI

To deal with uncertainty (and intractability), human minds rely on an adaptive toolbox of heuristics (Gigerenzer et al., 2011; Hertwig et al., 2013). Note that logic and probability theory cannot provide the necessary and sufficient tools for rational cognition under uncertainty because that would require well-defined and stable situations—small worlds. Instead, human minds rely on robust and efficient tools, such as heuristics based on recency, frequency, recognition, trust, and imitation, and on cognitive capacities, such as systematic forgetting (Gigerenzer et al., 2022). None of these mental tools are needed in small worlds, and they have consequently been confused with irrationality when experimental participants are tested on small-world problems (Gigerenzer, 2018).

In sum, I argue that genuine psychological processes that deal with uncertainty (as opposed to logic and probability theory) are likely useful for the design of algorithms. In what follows, I focus on one class of tasks that involves uncertainty: predicting future events.

## Recency: One Data Point Can Beat Big Data

Recency is the tendency to give more importance to recent events than to those of the past. In memory research, a “recency effect” has been observed, which occurs if events more recently encountered are remembered best. Recency is sometimes seen as a bias in the sense of error, as in the availability bias, but this valuation overlooks that recency, like all psychological phenomena, is neither rational nor irrational. Rather, one needs to assess its ecological rationality. In a stable world, relying on the most recent events only and ignoring the base rates (the data of the past) might indeed be an error. But in an unstable world, in which unexpected events happen, relying on recency may well lead to better decisions.

Consider the classical argument that people should rely on the long-term base rates and not be influenced by recent events. Nisbett and Ross (1980, p. 15) presented their readers a choice between purchasing either a Volvo or a Saab in which the sole criterion is the car’s life expectancy. The only information available is that among several hundred Volvos and Saabs, one Volvo and a dozen Saabs broke down. Just yesterday, a neighbor mentioned that his new Volvo broke down. Which car should one buy? According to Nisbett and Ross’s Bayesian argument, the neighbor’s recent report changes the record by only an iota, so buying the Volvo would be rational. That argument assumes a stable world and may well be correct in this case, unless a new generation of Volvos is systematically more flawed. Now consider a variation of the choice problem (Gigerenzer, 2000, p. 263): You are a parent who has to decide whether to let your child swim in a river or climb trees instead, and the sole criterion is the child’s life expectancy. It is known that among many hundred children, only one accident occurred in the river, whereas a dozen children had accidents falling from trees. Just yesterday, a neighbor said that her child was eaten by a crocodile in the river. Where would you send your child? Although Bayesian updating would indicate that sending the child to the river is a more rational choice, in this situation, there is good reason to suspect that the environment has changed. Here, relying on the most recent information and ignoring past data is likely ecologically rational.

This thought experiment illustrates the principle of ecological rationality: Relying on recency is not unconditionally irrational but can be ecologically rational in situations of uncertainty, whereas Bayesian updating is rational in a stable world of risk. Psychological research on recency dates back at least to Brown’s (1838) “law of recency,” which states that recent experiences come to mind more easily and are often the sole information guiding decisions. Likewise, studies on firefighter officers, military personnel, and handball players have reported that these experts tend to rely on the first option that comes to mind and that more time and deliberation tend to increase errors rather than decrease them (Johnson & Raab, 2003; Klein, 1998/2017). Consistent with the principle of ecological rationality, people tend to use recency adaptively, depending on the structure of the environment (Schooler & Anderson, 2016; Schooler & Hertwig, 2005).

The stable-world principle suggests one condition in which recency can be ecologically rational: situations that can change unexpectedly. Predicting future events is one such situation. Can the recency heuristic help build better algorithms for forecasting events?

### Predicting the flu

To indicate where the flu is spreading, Google engineers used big-data analytics to predict the rate of flu-related doctor visits on a daily or weekly basis. To design GFT, they analyzed 50 million queries submitted to the Google search engine and selected 45 variables from these, which they combined in a secret algorithm (Ginsberg et al., 2009). GFT was trained on data from 2003 to 2007 and tested on data from 2007 to 2008. Yet after the swine flu struck in the spring of 2009, GFT consistently underpredicted the flu-related doctor visits over months. It had learned that the flu is high in the winter and low in the summer, but the swine flu followed a different rhythm. To improve the algorithm, the engineers opted to make it more complex instead of simpler and increased the number of variables to approximately 160 (Cook et al., 2011). Additional complexity, however, did not improve the algorithm; the updated version now overestimated the proportion of flu-related doctor visits in 100 out of 108 weeks from August 2011 to September 2013, in some cases overshooting the actual visits by more than 50% (Butler, 2013). In response, the Google engineers once again suspected that their model was too simple and updated it once again in 2013 (Copeland et al., 2013). After yet another update in 2014, GFT was shut down a year later.

Flu viruses do not create a stable situation. Instead of betting on more data and increasing the complexity of the algorithm, the alternative approach is psychological AI. In fast-changing environments, it is rational to decrease complexity and ignore past data. Katsikopoulos et al. (2022b) designed the simplest version of the recency heuristic, which implements these principles and relies on a single data point. They programmed recency into a fast-and-frugal algorithm.

#### Recency heuristic

To predict that this week’s proportion of flu-related doctor visits equals the proportion from the most recent week.

One can intuitively see that this algorithm can quickly adapt to sudden and unexpected events such as the swine flu, whereas big data make it difficult to change course. The weekly predictions of the recency heuristic were tested from March 18, 2007, to August 9, 2015 (the same time as GFT made predictions). The overall error was measured by the average absolute difference between the predicted and observed values of flu-related doctor visits divided by the observed value (mean absolute prediction error). The mean error was 9% for the recency heuristic compared with 20% for GFT. Alternative measures of error also resulted in half the error for the recency heuristic compared with GFT (Katsikopoulos et al., 2022a). The advantage of the psychologically inspired algorithm held for every year between 2007 and 2015 and across all three updates of GFT. Here, one data point was more powerful than big data. This case is an existence proof of the benefits of psychological AI.

Recency and GFT’s big-data analytics can also be combined to generate hybrid algorithms (Lazer et al., 2014), but at the cost of transparency. Recency can also be implemented as the most recent “trend” (rather than point estimate) and have similarly accurate results (Katsikopoulos et al., 2022a).

Forecasting challenges, such as the Center for Disease Control and Prevention’s (CDC) FluSight, have shown that no single model can predict influence-like illnesses (ILI) best for all forecast horizons and locations. A few contests have used the recency heuristic as a benchmark. Aiken et al. (2021) tested a time-series-specific neural network (gated recurrent unit neural network), two regularized linear regression models (linear autoregression and linear network autoregression), and a random-forest model (with maximum tree depth selected by fourfold cross-validation to protect against overfitting). They reported that when the time horizon for prediction was 1 to 2 weeks (“nowcasts,” corresponding to the 1 to 2 weeks’ reporting delay of the CDC data), none of these models could predict better than recency. When the time horizon was 3 to 4 weeks (“forecasts”), the power of recency faded, and the neural network predicted best. In other words, the rationality of recency lies in the extent of its recency. Turner et al. (2022) studied weekly forecasts of ILI from February 2021 to May 2022, during which COVID-19 disrupted the spread of the flu—an unexpected event resembling the sudden emergence of the swine flu in 2009. They tested six models: an autoregressive neural network, unrestricted and restricted autoregressive integrated moving averages, unrestricted seasonal autoregressive integrated moving average, seasonal and nonseasonal simple exponential smoothing, and an ensemble model, which was the average of all six models. The models were trained on data either from October 2010 or from January 2020 (“long” and “short” data set) up to the date when the forecast was made, resulting in 14 models. The simplest model, exponential smoothing, predicted best. None of the other models could consistently predict ILI better than the recency heuristic, which served as benchmark. Curiously, the authors of both studies focussed on discussing the relative merits of complex models and paid little attention to the power of recency or to the question of how to integrate it into machine-learning models.

The potential of recency has been observed more generally in situations characterized by uncertainty. In economics, Dosi et al. (2020) showed that the recency heuristic predicted demand in volatile and disruptive environments better than did complex macroeconomic models. Recency has also been implemented in algorithms for predicting individuals’ smartphone usage behavior, which is not static but may change over time (Sarker et al., 2019). When forecasting future customer purchases in 27 retail businesses, recency was as accurate as random forests and logistic regression even though the latter used more data points (Artinger et al., 2018). Artinger et al. (2023) developed general conditions under which recency can predict future events at least as well as algorithms that integrate all available information and showed that if these conditions hold, predictions of future sport events and crime were as accurate as those made by random forests—and more transparent to boot.

## Rounding Can Increase Transparency With No Loss of Predictive Accuracy

Rounding precise numbers can be regarded as a loss of information. Yet humans communicate round numbers more frequently than precise numbers, including about time, frequency, or chance (Nguyen et al., 2022). For instance, when asked about time, people tend to respond with numbers rounded to the nearest 5-min or 15-min values even when their digital watch displays the exact time (van der Henst & Sperber, 2004). The loss of precision, however, enables faster recall and mental manipulation of time units (Solt et al., 2017). This positive effect is not observed only for time. Nguyen et al. (2022) presented their participants with three news articles that contained either precise or rounded numbers. For instance, in an article on applicants admitted to the University of California, the precise [rounded] snippet read: “Among the admitted freshmen were 28,752 [30,000] transfer students who were offered spots at U.C. campuses, out of 41,282 [40,000] applicants.” When participants read an article containing a rounded number, the average recalled number more closely approximated the precise number (which those participants had not seen) than in the case of participants who read an article containing the precise number. Rounding also led to better performance when participants were asked to estimate the percentage of transfer-student applicants who were offered spots (Nguyen et al., 2022). In the rounding condition, participants also responded more quickly. In sum, by decreasing the precision of numbers, one can increase the accuracy and speed of recall and estimation. These results have been interpreted in the context of relevance theory (Sperber & Wilson, 1986), according to which people communicate maximum relevance, not precision. Can rounding help build better algorithms for forecasting events?

### Predicting failure to appear at court

After defendants have been arrested in the United States, a judge must decide whether individuals, while awaiting trial, should be released on their own recognizance or subject to monetary bail. In practice, the latter often means jail for defendants who are too poor to post bail. To aid judges with these pretrial decisions, black-box algorithms are used that calculate risk scores for each defendant. For instance, the commercial risk-assessment tool COMPAS makes use of more than 130 (undisclosed) variables; because the algorithm is proprietary, we also do not know whether it is a linear multiple regression or some nonlinear combination of the variables. Although COMPAS has been consulted in U.S. courts for more than a million defendants, it cannot predict more accurately than poorly paid workers for Amazon Turk having little to no experience with the task (Dressel & Farid, 2018).

Predicting whether a defendant will appear in court is fraught with uncertainty. In this situation, it would be worthwhile to incorporate psychological principles for dealing with uncertainty into the construction of an algorithm. On the basis of the research described above, Jung et al. (2020) introduced the rounding principle, that is, sacrificing precision to improve prediction. The result was a select-regress-and-round method that combines this psychological insight with feature selection using machine-learning techniques to form a hybrid model:

Step 1: Select *k* features from the set of all features in a stepwise way by iteratively adding the feature that minimizes the Akaike information criterion.
Step 2: Regress: Using only these *k* features, train a L^1^-regularized (least absolute shrinkage and selection operator [lasso]) logistic regression model on the data, resulting in *k* fitted beta weights.
Step 3: Round the weights by rescaling them in a range [–M, M], where M is an integer, and then round the rescaled weights to the nearest integer.

Note that rounding may result in algorithms with fewer than *k* features because it may generate zero weights in Step 3. Jung et al. (2020) tested the resulting algorithms on 21 data sets from the UCI Machine Learning Repository and on 165,000 adult criminal-court cases involving nonviolent offenses. The authors reported that for the Machine Learning Repository data, simple algorithms with only five features and rounded (integer) weights in the range [–3, 3] performed on par with logistic regression and lasso regression despite the latter being trained on all features (range = 5–41). The court cases involved pretrial decisions, and the court data contained 64 features for each defendant, including characteristics of the charge (e.g., theft, gun-related) and the defendant (e.g., gender, age). The task was to predict a defendant’s flight risk, that is, the failure to appear at required court proceedings. The select-regress-and-round method resulted in a simple algorithm that used only two features, age and number of earlier failures to appear. The rounded weights are as follows:

*Age:   Prior Failures to Appear (FTA)*

18 ≤ age < 21 Score: 3  1 prior FTA Score: 2

21≤ age < 31 Score: 2  2 or more FTAs Score: 3

31≤ age < 51 Score: 1

The risk value for a defendant is the sum of the values on the two features. The final decision to release or bail must be based on a threshold: 3.5 in the current study. For example, a 25-year-old with no prior failures to appear has a risk score of 2 and thus would be released. Jung et al. (2020) reported that this simple algorithm matched complex machine-learning models that used all 64 features and outperformed professional human judges.

The idea of rounding weights before making a prediction is not new but has had a short and neglected history. Drawing on the work by Dawes and Corrigan (1974), Einhorn and Hogarth (1975) introduced equal-weight linear models, such as rounding all negative weights to –1 and all positive ones to +1. This seminal work attracted little attention among researchers who routinely rely on regression analysis featuring precise beta weights. Hogarth (2012) reported that standard textbooks in econometrics teach students neither about the unreliability of precise regression weights nor about equal-weight algorithms as a general benchmark for prediction. Czerlinski et al. (1999) tested linear models in out-of-sample prediction in 20 forecasting tasks and found that, on average, using rounded weights (+1 or –1) led to better predictions than did multiple linear regression (using precise beta weights). On the basis of this work, the Bank of England tested the predictive value of its risk-based models and similarly found that rounded weights (e.g., the equal weights in leverage ratios) predicted bank failure more accurately than the standard risk-weighted models in the Basel-III regulation (Aikman et al., 2021; Haldane & Madouros, 2012). Katsikopoulos et al. (2020) analyzed in more detail the ecological rationality of rounded weights in algorithms known as “tallying” and compared their performance with ridge and lasso-regression models, random forests, and other standard machine-learning techniques. Tallying simply weights all variables equally, thereby avoiding estimation error at the cost of potentially increasing bias. The situations that support tallying include substantial uncertainty (e.g., out-of-population prediction vs. out-of-sample prediction), the absence of dominant cues (which is an environment recency can exploit), and conditions such as scarce data relative to the number of predictors (Katsikopoulos et al., 2020). In machine learning, a related idea to reduce model complexity and prevent overfitting is regularization, as in ridge and lasso regression. Consider predicting the survival of patients with bladder, breast, and ovarian cancer out of population, that is, by algorithms trained on one population and applied to another, as is common in health care. Simply setting all weights to be equal performed as well as ridge and lasso regressions (see Katsikopoulos et al., 2020). The advantage of equal weights appears to be largest in out-of-population prediction, such as when an algorithm is developed in one hospital and applied in others.

## Explainable AI

Many machine-learning models for high-stakes decisions, such as in criminal justice and health care, tend to be black-box models. They are either too complicated to be understood by their users, proprietary business secrets, or both. For instance, GFT has been criticized for its lack of transparency: The variables used were not revealed publicly, nor were the details of the three model updates (Lazer et al., 2014).

Nontransparent algorithms have encountered resistance and hinder the mass adoption of AI systems for several reasons. Physicians, for instance, may be hesitant to follow treatment recommendations of a black-box algorithm whose logic is opaque. Health authorities became concerned after machine-learning-based models made severe errors, such as recommending highly polluted air as safe to breathe (see Rudin, 2019). Members of minorities may suspect that prejudices against them are hidden away in COMPAS’s black box or in predictive policing software.

In reaction to this acceptance problem, work on explainable AI (XAI) emerged. Yet for the most part, the black box was retained and supposedly explained by a different model that was created post hoc. In the case of deep artificial neural networks, which are black boxes because they are highly recursive, heat maps and saliency maps have been consulted to visualize where the network is “looking,” yet the resulting explanations may or may not reflect what the AI is actually doing (Ghassemi et al., 2021).

### The myth of the accuracy-interpretability trade-off

The insistence on complex black-box models has been justified by the purported “accuracy-interpretability trade-off”: The more accurate an algorithm is, the less interpretable or understandable it is. For instance, the Defense Advanced Research Projects Agency (2016) Explainable AI program published a widely circulated figure that shows this trade-off, in which the interpretability of various algorithms decreases with its accuracy. This figure, however, appears not to be based on actual data (the axes are not even quantified) but on a blind belief in the existence of such a trade-off (Rudin, 2019). As the case of flu predictions and recidivism predictions illustrate, this trade-off does not generally exist; interpretable algorithms can often predict as well or better than complex ones (Gigerenzer et al., 2011; Rudin & Radin, 2019). After reviewing 97 studies that compared simple and complex forecasting methods, Green and Armstrong (2015) reported that complexity increased forecast error by 27% on average. The real question is when this can happen, and the stable-world principle provides a first answer: in ill-defined problems that are not stable over time. Here, accuracy can actually increase with transparency.

Psychological AI opens up a new vision for XAI. Instead of seeking to explain opaque complex systems, researchers should first investigate whether psychological AI offers a transparent and equally accurate solution. If so, as in the case of predicting the flu and flight risk, the explainability problem is solved. There is a growing body of research in machine learning that investigates understandable rules such as short decision lists (Certifiably Optimal RulE ListS; see Angelino et al., 2018), simple decision trees, and regularization, as in lasso and ridge regression (Khaleghi, 2019). Simple algorithms are more likely to be understandable and simultaneously protect researchers against overfitting. It should become routine to first create algorithms that can be easily understood and explained correctly and use these as a benchmark to empirically evaluate whether more complexity in fact increases accuracy substantially (Katsikopoulos et al., 2020).

## A Research Agenda

In this article, I posed two questions. First, is there any use for psychology in the design of AI algorithms? As the two cases studies illustrate, the answer is “yes.” Second, what kind of psychological insights are helpful? I argue that these are the heuristic principles that humans rely on when dealing with uncertainty. By exploiting these, psychological AI can increase both predictive power and transparency.

I focused on designing algorithms for predicting future events. Yet psychological insights can be useful for other purposes. For instance, psychological research (Rieskamp & Otto, 2006) has shown that people intuitively rely on the take-the-best heuristic in tasks in which its use is ecologically rational. The latter is the case when at least one of three conditions holds: cue weights decrease exponentially (“noncompensatoriness”), exhibit “cumulative dominance,” or exhibit “simple dominance.” ŞimŞek (2013) found that these conditions are prevalent in a large, diverse collection of machine-learning data sets, which makes the limited search embodied in heuristics rational. ŞimŞek et al. (2016) detected that the same structures are prevalent in the approximately 1.6 × 10^60^ possible states of the computer game Tetris. This insight allows Tetris’s search space to be reduced dramatically and enables development of faster and more effective learning algorithms. The case of Tetris is particularly interesting because it amounts to an application of psychological AI to a well-defined but intractable game. That indicates that the core domain of psychological AI might be broader than I assume here.

Finally, psychological AI contributes to the literature on XAI. Unlike black-box models, it can deliver algorithms that are understandable, explainable, and easily adaptable to new situations. The General Data Protection Regulation of the European Union prescribes transparent information and processing of personal data. Transparency is an important value in democratic societies in the process of being digitalized. Important decisions about health, wealth, and bail should be understandable to individuals who have to bear the consequences.

## Transparency

*Action Editor:* Sudeep Bhatia

*Editor:* Interim Editorial Panel
